# Effects of Tannic Acid on Immune Function and Gut Microbiota in Brandt’s Voles (*Lasiopodomys brandtii*)

**DOI:** 10.3390/microorganisms14030577

**Published:** 2026-03-03

**Authors:** Jin Li, Kunying Zhou, Di Xu, Yunqi Liu, Yu Sun, Deli Xu

**Affiliations:** School of Life Sciences, Qufu Normal University, Qufu 273165, China; 17861824275@163.com (J.L.); zky7665@163.com (K.Z.); xdvin458@163.com (D.X.); 17562002639@163.com (Y.L.); 13555316428@163.com (Y.S.)

**Keywords:** Brandt’s vole, tannic acid, polyphenols, gut microbiota, immunity, host–microbiota interaction, dose–response

## Abstract

The present study investigates the effects of tannic acid (TA) on body composition, immune function, and gut microbiota in Brandt’s voles (*Lasiopodomys brandtii*); analyzes the gut microbiota–immune parameter associations during their response to plant secondary metabolites; and provides a theoretical basis for understanding their adaptive mechanisms. Thirty-three female Brandt’s voles were randomly divided into four groups and intragastrically administered distilled water (control group) or TA at doses of 300, 600, and 1200 mg·kg^−1^·d^−1^ for 9 weeks. The results showed that TA had no significant effect on body mass, body composition (including subcutaneous, retroperitoneal, mesenteric, and perigonadal fat, as well as total fat mass), immune organ weights, or cellular immune responses in Brandt’s voles. However, high-dose TA (1200 mg·kg^−1^·d^−1^) significantly reduced the serum anti-KLH IgG titers in a dose-dependent manner, indicating selective impairment of humoral immunity. High-dose TA (1200 mg·kg^−1^·d^−1^) also decreased the alpha diversity of the gut microbiota, with lower Chao1, Observed features, and Shannon indices compared to the control and low-dose (300 mg·kg^−1^·d^−1^) groups. Beta diversity analysis indicated that high-dose TA (1200 mg·kg^−1^·d^−1^) altered the overall gut microbiota structure, while taxonomic analyses revealed a decrease in Desulfobacterota and an increase in several gut-associated taxa, including Firmicutes, Clostridia, Lachnospirales, and Lachnospiraceae. In conclusion, high-dose TA (1200 mg·kg^−1^·d^−1^) induced significant changes in the gut microbiota and selectively suppressed humoral immunity. However, other immune parameters and growth-related measures remained unaffected. These findings suggest a potential role of gut microbial adjustments in modulating host responses to dietary TA and contribute to knowledge of the tolerance mechanisms in this species.

## 1. Introduction

Under normal physiological conditions, the gut microbiota fulfills essential roles in host digestion, intestinal homeostasis, and immune regulation [1,2,3,4]. Diet is a major environmental factor influencing gut microbiota composition [1,5,6], and immune regulation is also affected by food quality [7,8].

Polyphenols are widely distributed in fruits, vegetables, grains, tea, and medicinal plants [9], among which tannic acid (TA), a ubiquitous polyphenolic compound, exhibits anti-inflammatory, antioxidant, antibacterial, and neuroprotective properties [10,11]. TA can form complexes with proteins, starch, and digestive enzymes, making it one of the most common antinutritional factors in food and potentially reducing nutrient digestibility and growth performance [12]. Consistent with these dual properties, it has been shown to regulate inflammatory processes and gastrointestinal barrier function in a dose-dependent manner [13,14]. At relatively low concentrations, TA can improve intestinal morphology, modulate gut microbiota composition, and enhance intestinal barrier function in laboratory animals, including C57BL/6 mice [9], weaned piglets [15,16], and broiler chickens [14,17,18,19]. In contrast, high-dose TA has been reported to impair antioxidant capacity and disrupt intestinal barrier integrity, as demonstrated in broiler chickens exposed to 5000 mg/kg of TA [14]. Similarly, short-term intake of high-dose TA has been shown to impair intestinal immune function, alter gut microbiota composition, and compromise intestinal physiology in male Brandt’s voles [20,21]. Despite these advances, studies have largely focused on short-term exposure and predominantly on male individuals. Accumulating evidence suggests that host responses to dietary polyphenols, including TA, may exhibit sex-specific physiological and microbial differences [22], but experimental data on the long-term effects of TA in female wild small herbivorous mammals remain scarce. This limitation constrains our understanding of how plant secondary metabolites influence host tolerance and adaptation under natural dietary conditions and highlights the need for targeted investigation in females under prolonged exposure.

Brandt’s vole (*Lasiopodomys brandtii*) is a small rodent species predominantly distributed in the typical steppe regions of Inner Mongolia, eastern Mongolia, and the Transbaikal region of Russia [23]. During years of high population density, it poses a significant threat to the grassland grazing industry and crop production [24]. *Leymus chinensis* is one of the primary forage plants of Brandt’s voles and contains approximately 2.8 mg of tannins per kilogram. Based on the average tannic acid concentration in *L. chinensis* and field observations of food intake by Brandt’s voles, a wild vole weighing 50 g can ingest 0.030–0.069 g of tannic acid per day [25,26]. Accordingly, Brandt’s vole represents a suitable ecological model for examining dose-dependent and long-term effects of plant polyphenols under naturalistic dietary exposure scenarios.

Despite this ecological relevance, how female Brandt’s voles respond to chronic, quantitatively defined TA exposure at the level of the gut microbiota–immune axis remains poorly understood. We therefore hypothesized that (i) TA exposure would induce dose-dependent alterations in gut microbiota diversity and community structure; (ii) TA exposure would exert dose-dependent effects on host immune parameters; and (iii) shifts in microbial composition are associated with variations in host immune parameters. Therefore, this study employed microbiome sequencing to investigate the impacts of TA (at doses not exceeding the average content in the typical diet) on body composition, immune function, and gut microbial structure and function in female Brandt’s voles from the perspective of the gut microbiota. By integrating ecologically relevant dosing with sex-specific investigation, this study aims to provide new insights into the adaptive strategies of female Brandt’s voles in response to plant secondary metabolites and to clarify dose-dependent interactions among dietary components, gut microbiota, and immunity.

## 2. Materials and Methods

### 2.1. Experimental Design

All animal procedures were conducted in accordance with protocols approved by the Animal Care and Use Committee of Qufu Normal University (Approval No. 2021013). Brandt’s voles were individually housed in plastic cages (30 cm × 15 cm × 20 cm) with sawdust as the bedding material, under a controlled 12 h:12 h light–dark photoperiod and a constant temperature of 23 ± 1 °C. Throughout the experiment, all animals were provided with standard rat pellet chow (Beijing KeAo Feed Co., Beijing, China) and tap water ad libitum.

The estimated daily TA intake for a wild Brandt’s vole (approximately 50 g body mass) ranged from 0.030 to 0.069 g, based on the TA concentration in its preferred plants (e.g., *Stipa krylovii*, *Leymus chinensis*, and *Cleistogenes squarrosa*) and its field consumption [25,27]. Accordingly, the experimental doses used in this study (300, 600, and 1200 mg·kg^−1^·d^−1^, respectively) were selected to represent low, intermediate, and high levels within this estimated natural intake range, enabling the assessment of dose-dependent physiological and microbial responses under ecologically relevant exposure conditions. Brandt’s voles were initially randomized into four groups (*n* = 9 per group). Following a 30-day acclimation period, to reduce overall sample heterogeneity, two voles (one control and one low-dose) were excluded due to body mass deviations exceeding 10%. Additionally, one vole from the low-dose group was excluded during the treatment period due to unrelated health issues and was not included in the final analysis. All exclusions were random and not influenced by the experimental treatments, and after these exclusions, the final sample sizes were as follows: Control (*n* = 8), Low (*n* = 7), Mid (*n* = 9), and High (*n* = 9). Daily oral gavage (0.2 mL) of TA (0, 300, 600, and 1200 mg·kg^−1^·d^−1^) was given for 63 consecutive days (9 weeks) to simulate chronic dietary exposure over a substantial portion of the growing and foraging season, reflecting long-term ecological pressure rather than acute TA intake. Gavage was performed within a fixed time window (15:00–17:00), and body mass was recorded daily. The administered volume was adjusted according to body mass to ensure a constant TA dose (mg·kg^−1^·d^−1^) across individuals. The control group received an equivalent volume of distilled water via gavage to standardize procedural and circadian effects across treatments. Humoral immunity was assessed by subcutaneous injection of keyhole limpet hemocyanin (KLH; Sigma, Tokyo, Japan, LH7017) 10 days prior to euthanasia, a time point corresponding to the peak production phase of IgG in rodents’ immune response to KLH, ensuring the precise capture of maximum IgG secretion levels [28,29]. Cell-mediated immunity was evaluated via a phytohemagglutinin (PHA) response 3 days before euthanasia to reflect the long-term effects of TA treatment on cellular immunity [30,31,32]. At the end of the experiment, all voles were euthanized by CO_2_ asphyxiation in accordance with AVMA guidelines, and death was confirmed by the absence of respiration and cardiac arrest, followed by cervical dislocation [33]. Immediately after euthanasia, the cecum, spleen, and thymus were dissected, weighed, and snap-frozen at −80 °C for subsequent analysis, and trunk blood was collected and centrifuged to separate serum for quantifying IgG titers. For microbiota analyses, seven samples per group were randomly selected for 16S rRNA gene sequencing to ensure balanced group sizes and consistent sequencing depth across treatments, while maintaining sufficient statistical power for community-level comparisons.

### 2.2. Body Composition

All visceral organs were removed to obtain the wet carcass mass [34]. Subsequently, the fat deposits—including subcutaneous, retroperitoneal, mesenteric, and perigonadal fat—were carefully dissected and weighed. The combined weight of these four fat types was defined as the total body fat mass [34]. The percentage of subcutaneous, retroperitoneal, mesenteric, perigonadal, and total body fat was calculated by dividing each respective fat mass by the wet carcass mass [35], in accordance with established protocols in rodent physiological ecology, which used wet mass as the denominator to express fat reserves as a proportion of the animal’s total physiological mass, ensuring both ecological relevance and comparability with previous studies [32].

### 2.3. Cellular Immunity Assays

Cellular immunity was detected by injecting exogenous immune-stimulating substances (phytohemagglutinin, PHA) [36,37]. Three days before euthanasia, the initial footpad thickness of each vole was determined by averaging six measurements taken with a digital micrometer (547-301 Absolute Digimatic Indicator ID-C, Mitutoyo Co., Kawasaki, Japan). Immediately thereafter, each vole received a subcutaneous injection of 0.1 mg of PHA (PHA-P, Sigma L-8754) in 0.03 mL of sterile saline (pH 7.4) into the center of the footpad. Footpad thickness was remeasured at 6, 12, 24, and 48 h post injection, with six measurements averaged per time point [30,38,39]. The PHA response, representing cellular immunity, was calculated as (post-injection thickness—initial thickness)/initial thickness [30,31].

### 2.4. Humoral Immunity Assays

Humoral immunity was evaluated by measuring serum anti-KLH IgG titers. To this end, Brandt’s voles received a single subcutaneous injection of 100 μg of KLH (Sigma LH7017) in 0.1 mL of sterile saline 10 days before euthanasia. Trunk blood was collected, clotted on ice for 1 h, and centrifuged at 4000 rpm (4 °C, 30 min) to obtain serum. Anti-KLH IgG titers were quantified using a standard enzyme-linked immunosorbent assay (ELISA) according to established methods [28,29].

Briefly, 96-well microplates were coated with 100 µL per well of KLH (0.5 mg mL^−1^) dissolved in 0.05 mol L^−1^ of Na_2_CO_3_–NaHCO_3_ buffer (pH 9.6), and incubated overnight at 4 °C. The plates were washed three times with PBS-T (phosphate-buffered saline containing 0.05% Tween 20, pH 7.4) and then blocked with 100 µL per well of 5% non-fat dry milk in PBS-T at 4 °C overnight to reduce non-specific binding. After removing the blocking solution, the plates were washed three times with PBS-T. Serum samples were diluted 1:20 in PBS-T, and 150 µL of each diluted sample was added to the antigen-coated wells. Positive and negative control samples (pooled sera from voles repeatedly challenged with KLH and from KLH-naïve voles, respectively) were similarly diluted in PBS-T and added in duplicate. The plates were sealed and incubated at 37 °C for 3 h, then washed three times with PBS-T. Alkaline phosphatase-conjugated anti-mouse IgG secondary antibody (1:2000 in PBS-T; Sigma) was added (100 µL per well) and incubated for 1 h at 37 °C. After washing, 150 µL of p-nitrophenyl phosphate substrate solution (1 mg mL^−1^ in diethanolamine buffer; Sigma) was added to each well, and the enzyme–substrate reaction was incubated in the dark at 37 °C for 20 min. The reaction was terminated by adding 50 µL of 1.5 mol L^−1^ NaOH. Absorbance was measured at 405 nm using a microplate reader (Bio-Rad Benchmark, Richmond, CA, USA), with each sample analyzed in duplicate. Due to the absence of a suitable standard, optical density (OD) values were used to represent the relative levels of anti-KLH antibodies [28,29].

### 2.5. DNA Extraction and PCR Amplification

Genomic DNA was extracted from the intestinal contents of Brandt’s voles using the cetyltrimethylammonium bromide (CTAB) method. The concentration and purity of the extracted DNA were assessed by electrophoresis on 1% agarose gel. Based on DNA concentration, samples were diluted with sterile deionized water to a final concentration of 1 ng/μL. The V3–V4 region of the 16S rRNA gene was amplified using the universal primer set 341F and 806R with sample-specific barcodes. This primer pair provides broad coverage of major bacterial phyla commonly found in mammalian gut microbiota, including Firmicutes, Bacteroidota, Proteobacteria, and Actinobacteriota, and has been experimentally validated to yield taxonomic profiles comparable to PCR-free metagenomic data, indicating relatively low amplification bias [40]. PCR products were purified using magnetic bead purification and then mixed with an equal volume of 1× TAE buffer for electrophoretic detection on 2% agarose gel. Sequencing libraries were constructed using the NEBNext^®^ Ultra™ II DNA (New England Biolabs, Ipswich, MA, USA) Library Prep Kit, and library quality was assessed using Qubit fluorometry and quantitative PCR (Q-PCR). Qualified libraries were subsequently sequenced on the Illumina NovaSeq platform using paired-end sequencing [41,42].

### 2.6. Bioinformatics Analysis

Paired-end reads were assigned to samples based on their unique barcodes and truncated by removing barcode and primer sequences [43]. Demultiplexed sequences from each sample were quality-filtered, trimmed, denoised, and merged, after which chimeric sequences were identified and removed using the DADA2 modules implemented in QIIME2 (2025. 04) to obtain amplicon sequence variants (ASVs) [44].

Taxonomic annotation of each ASV was performed using the QIIME2 classify-sklearn algorithm [45]. Bar charts were generated using QIIME2 plug-ins to visualize differences in taxonomic composition among samples at each taxonomic level. In addition, the top 10 taxa with the highest relative abundance were selected to generate ternary plots, allowing visual comparison of dominant taxa among different groups [46].

QIIME2 was used to calculate alpha diversity indices, including observed OTUs, Shannon, Simpson, Chao1, and Good’s coverage, to evaluate gut microbiota richness and diversity across groups. Beta diversity distance metrics, including Bray–Curtis dissimilarity, were calculated to assess structural variation in microbial communities among samples and were visualized using principal coordinate analysis (PCoA).

The *adonis* and *anosim* functions implemented in QIIME2 were applied to test for significant differences in community structure among the four groups. To identify microbial biomarkers, LEfSe was performed using the LEfSe (version 1.1.01) software with a stringent cutoff (LDA > 4) to reduce false positives and focus on taxa with larger effect sizes. Statistical significance was assessed using the Kruskal–Wallis test in LEfSe. Because LEfSe does not apply multiple-testing correction by default, these results were interpreted as exploratory, and emphasis was placed on taxa showing consistent group enrichment and large effect sizes. MetaStat analysis was conducted using R software (version 4.0.3) to identify taxa exhibiting significant differences among groups. In addition, putative functional profiles of microbial communities based on KEGG Orthologs (KOs) were inferred using Tax4Fun, which predicts functional potential from 16S rRNA gene-based taxonomic profiles.

### 2.7. Correlation Analysis

To analyze the role of gut microbiota in immune function, Pearson correlation analysis was performed between microbiota relative abundance (phylum and genus levels) and immune indices.

### 2.8. Statistical Analysis

Statistical analyses were performed using SPSS 26.0 and GraphPad Prism 10.1.2. Since body mass and PHA response were measured at multiple time points, repeated-measures ANOVA was used to analyze their temporal dynamics. Group differences in thymus and spleen mass were assessed using a General Linear Model (GLM) with final body mass included as a covariate, followed by Bonferroni post hoc tests, to account for allometric scaling between organ and body mass. One-way ANOVA with Tukey’s post hoc test was applied to all other data (body composition, IgG levels, and single-time-point comparisons of body mass/PHA response). Results are expressed as mean ± SE and a *p*-value < 0.05 was considered statistically significant. To quantify the magnitude of treatment effects, effect sizes are reported as η^2^ for one-way ANOVA and partial η^2^ (ηp^2^) for repeated-measures ANOVA and GLM analyses.

## 3. Results

### 3.1. Body Mass

**Figure 1:**
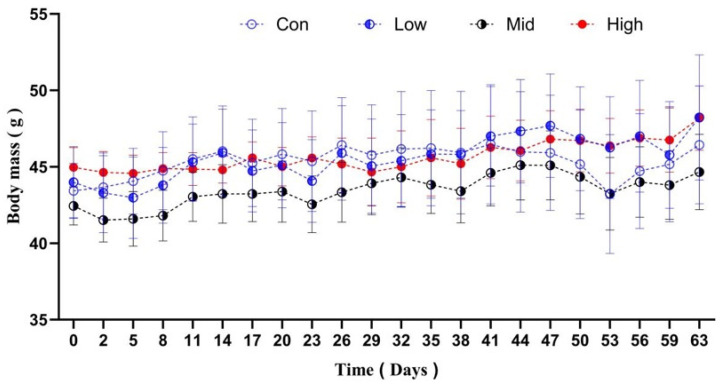
Effect of TA on body mass in Brandt’s voles. Con, control group; Low, low-dose TA group (300 mg·kg^−1^·d^−1^); Mid, medium-dose TA group (600 mg·kg^−1^·d^−1^); High, high-dose TA group (1200 mg·kg^−1^·d^−1^).

The voles’ body masses changed significantly over time (F_22,638_ = 3.094, *p* < 0.001, ηp^2^ = 0.098), but there was no significant time × group interaction (F_66,638_ = 0.382, *p* = 1.0, ηp^2^ = 0.040). No significant differences in body mass were observed among the groups on day 0 (F_3,29_ = 0.448, *p* = 0.720, η^2^ = 0.018) or throughout the experimental period, from day 2 (F_3,29_ = 0.548, *p* = 0.653, η^2^ = 0.025) to day 63 (F_3,29_ = 0.311, *p* = 0.817, η^2^ = 0.021) (Figure 1).

### 3.2. Body Composition

None of the body composition parameters, including wet carcass weight, as well as subcutaneous, mesenteric, retroperitoneal, perigonadal, and total body fat masses and their respective percentage contents, were affected by TA (Table 1).

### 3.3. Immune Organs

**Figure 2:**
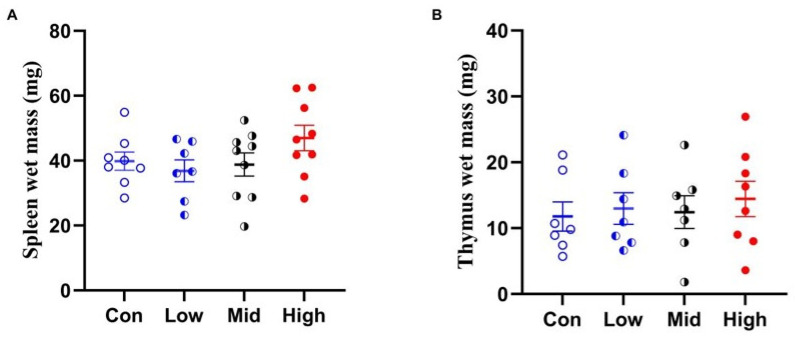
Effects of TA on the wet masses of spleen (**A**) and thymus (**B**) in Brandt’s voles. Con, control group; Low, low-dose TA group (300 mg·kg^−1^·d^−1^); Mid, medium-dose TA group (600 mg·kg^−1^·d^−1^); High, high-dose TA group (1200 mg·kg^−1^·d^−1^).

TA administration did not significantly affect the wet mass of either the spleen (F_3,28_ = 1.651, *p* = 0.200, ηp^2^ = 0.150) or the thymus (F_3,28_ = 0.464, *p* = 0.710, ηp^2^ = 0.047) in Brandt’s voles (Figure 2).

### 3.4. Cellular Immunity

**Figure 3:**
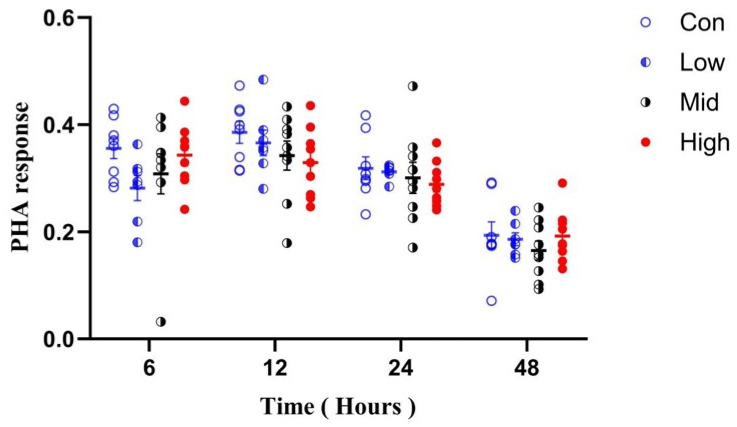
Effects of TA on cellular immunity in Brandt’s voles. Con, control group; Low, low-dose TA group (300 mg·kg^−1^·d^−1^); Mid, medium-dose TA group (600 mg·kg^−1^·d^−1^); High, high-dose TA group (1200 mg·kg^−1^·d^−1^).

For simplicity, PHA 6 h, 12 h, 24 h, and 48 h stand for PHA response at 6, 12, 24, and 48 h after PHA injection, respectively. It showed significant changes with treatment time (F_3,87_ = 103.672, *p* < 0.001, ηp^2^ = 0.771), but was not affected by the time × group interaction (F_9,87_ = 1.821, *p* = 0.076, ηp^2^ = 0.146). PHA responses at 6 h (F_3,29_ = 1.484, *p* = 0.240, η^2^ = 0.136), 12 h (F_3,29_ = 1.163, *p* = 0.341, η^2^ = 0.107), 24 h (F_3,29_ = 0.420, *p* = 0.740, η^2^ = 0.048), and 48 h (F_3,29_ = 0.544, *p* = 0.656, η^2^ = 0.032) were all unaffected by TA (Figure 3).

### 3.5. Humoral Immunity

**Figure 4:**
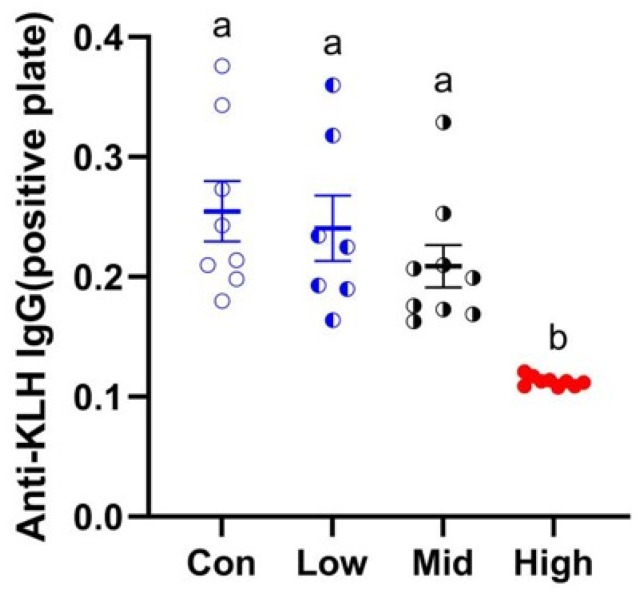
Effects of TA on serum anti-KLH IgG titers in Brandt’s voles. Different letters above the columns indicate statistical significance among the groups. Con, control group; Low, low-dose TA group (300 mg·kg^−1^·d^−1^); Mid, medium-dose TA group (600 mg·kg^−1^·d^−1^); High, high-dose TA group (1200 mg·kg^−1^·d^−1^).

TA treatment resulted in a dose-dependent decrease in serum IgG titers in Brandt’s voles, with the Low group showing a 5.52% reduction, the Mid group an 18.01% decrease, and the High group a 55.66% reduction compared to the control group, with the High group exhibiting significantly lower titers than all other groups (F_3,29_ = 11.293, *p* < 0.001, η^2^ = 0.539) (Figure 4).

### 3.6. Effect of TA on Colonic Gut Microbiota in Brandt’s Voles

#### 3.6.1. Species Annotation

**Figure 5:**
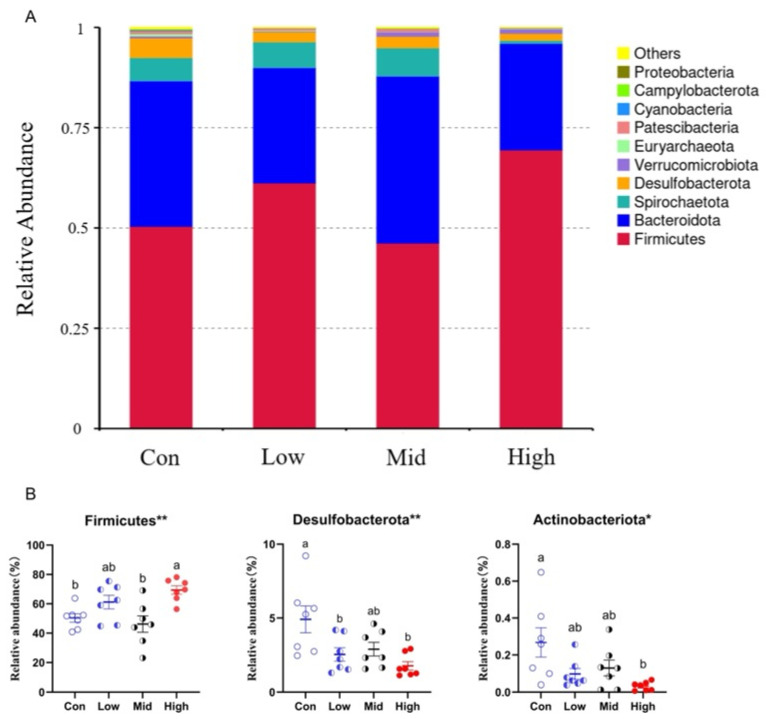
Effects of TA on gut microbiota at the phylum level in Brandt’s voles. (**A**) Relative abundance of gut microbiota at the phylum level. (**B**) Significantly different phyla among TA treatment groups. Con, control group; Low, low-dose TA group (300 mg·kg^−1^·d^−1^); Mid, medium-dose TA group (600 mg·kg^−1^·d^−1^); High, high-dose TA group (1200 mg·kg^−1^·d^−1^). For microbiota analyses, *n* = 7 samples per group. Group differences were assessed using one-way ANOVA. One star (*) indicates 0.01 ≤ *p* < 0.05, two stars (**) stand for 0.001 ≤ *p* < 0.01.

A total of 19 phyla, 28 classes, 58 orders, 72 families, 141 genera, and 42 species were annotated in the gut microbiota of Brandt’s voles. The dominant phyla included Firmicutes, Bacteroidota, Spirochaetota, Desulfobacterota, and Verrucomicrobiota (Figure 5A); the dominant families included Lachnospiraceae, Muribaculaceae, Bacteroidaceae, Ruminococcaceae, and Oscillospiraceae (Figure S1A); and the dominant genera included *Bacteroides*, *Lachnospiraceae_NK4A136_group*, *unidentified_Ruminococcaceae*, *Roseburia*, and *Hungatella* (Figure S1B).

At the phylum level, the relative abundance of Firmicutes was significantly higher in the High group than in the Con and Mid groups (F_3,24_ = 6.395, *p* = 0.002); similarly, that of Desulfobacterota was elevated in both the Low and High groups relative to the Con group (F_3,24_ = 5.451, *p* = 0.005). In contrast, the High group exhibited a significantly lower abundance of Actinobacteriota compared to the Con group (F_3,24_ = 4.380, *p* = 0.014) (Figure 5B). At the family level, the relative abundance of Lachnospiraceae in the High group was significantly higher than that in the other three groups (F_3,24_ = 12.289, *p* < 0.001); that of Desulfovibrionaceae in the Low and High groups was significantly lower than that in the Con group (F_3,24_ = 5.419, *p* = 0.005); and those of F082 (F_3,24_ = 3.595, *p* = 0.028), Marinifilaceae (F_3,24_ = 3.137, *p* = 0.044), and Eggerthellaceae (F_3,24_ = 3.250, *p* = 0.039) in the High group were all significantly lower than those in the Con group (Figure S2A–E). At the genus level, specifically, TA increased the relative abundances of *Lachnospiraceae_UCG-006* (F_3,24_ = 4.324, *p* = 0.014), *GCA-900066575* (F_3,24_ = 8.343, *p* = 0.001), and *[Eubacterium]_ruminantium_group* (F_3,24_ = 3.435, *p* = 0.033). In contrast, it reduced those of *Desulfovibrio* (F_3,24_ = 5.201, *p* = 0.007), *Enterorhabdus* (F_3,24_ = 3.593, *p* = 0.028), *Family_XIII_UCG-001* (F_3,24_ = 4.830, *p* = 0.009), *UBA1819* (F_3,24_ = 4.425, *p* = 0.013), *Treponema* (F_3,24_ = 3.263, *p* = 0.039), and *Odoribacter* (F_3,24_ = 3.387, *p* = 0.034) (Figure S2F–M).

#### 3.6.2. Diversity of Gut Microbiota

**Figure 6:**
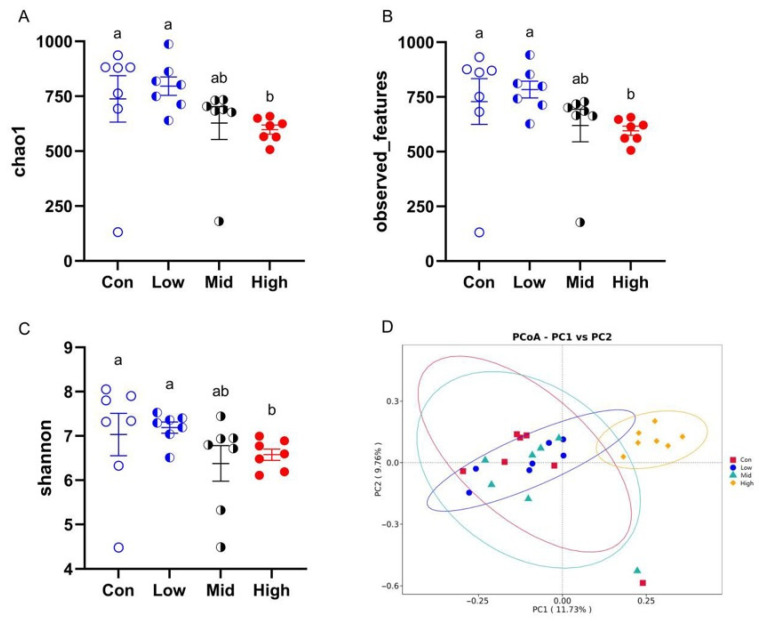
Effects of TA on the alpha and beta diversity of gut microbiota in Brandt’s voles. (**A**) Chao1 index. (**B**) Observed-features index. (**C**) Shannon index. (**D**) PCoA based on Bray–Curtis distance. Different letters above bars indicate significant differences among groups. Con, control group; Low, low-dose TA group (300 mg·kg^−1^·d^−1^); Mid, medium-dose TA group (600 mg·kg^−1^·d^−1^); High, high-dose TA group (1200 mg·kg^−1^·d^−1^). For microbiota analyses, *n* = 7 samples per group. Alpha diversity indices were compared using one-way ANOVA, and beta diversity was assessed using PERMANOVA (Adonis) and ANOSIM.

As determined by the Kruskal–Wallis test, gastric administration of high-dose TA (1200 mg·kg^−1^·d^−1^) significantly reduced the alpha diversity of the gut microbiota in Brandt’s voles. Specifically, the Chao1 (*p* < 0.001), Observed features (*p* < 0.001), and Shannon (*p* = 0.031) indices were all lower in the high-dose group (1200 mg·kg^−1^·d^−1^) compared to the Con and Low groups (Figure 6A–C). Principal coordinate analysis (PCoA) based on Bray–Curtis distance revealed a clear separation between the High group and the Con, Low, and Mid groups (Figure 6D). This pattern was supported by pairwise PERMANOVA (Adonis), which showed that the High group differed significantly from the Con (R^2^ = 0.169, *p* < 0.001), Low (R^2^ = 0.160, *p* < 0.001), and Mid (R^2^ = 0.147, *p* < 0.001) groups, whereas no significant differences were detected among the Con, Low, and Mid groups (R^2^ = 0.071–0.078, all *p* > 0.05). Consistently, pairwise ANOSIM indicated moderate to strong separation between the High group and the other treatment groups (R = 0.49–0.66, *p* ≤ 0.002), while negligible separation was observed among the Con, Low, and Mid groups (R ≈ 0, *p* > 0.5). Detailed test statistics and exact *p* values are provided in Table S1.

#### 3.6.3. Intergroup Differential Taxa Analysis

**Figure 7:**
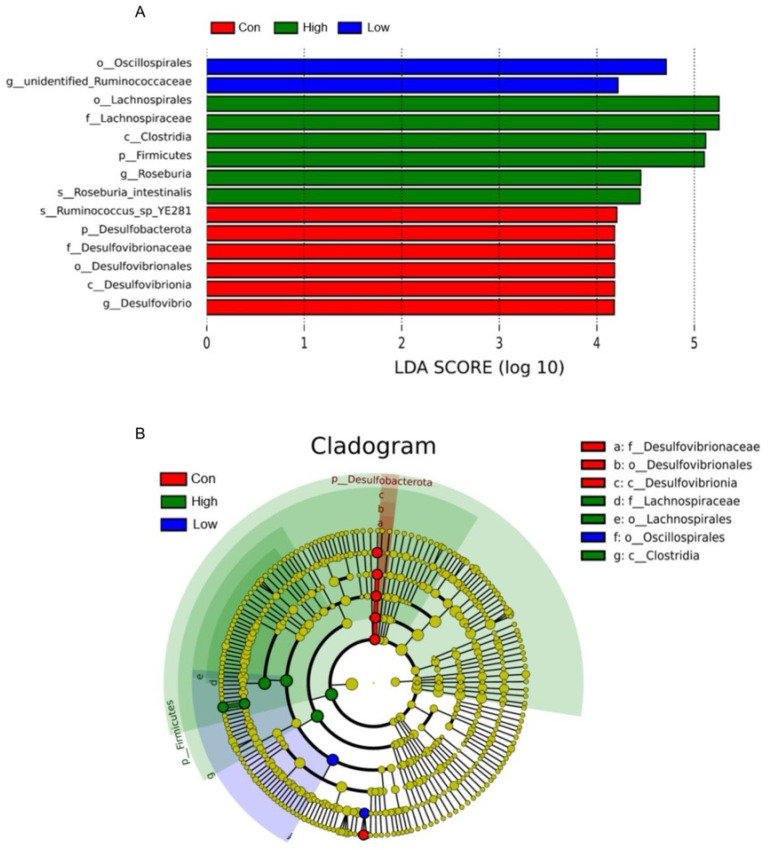
Effects of TA on gut microbiota LDA bar plot (**A**) and cladogram (**B**) in Brandt’s voles. Con, control group; Low, low-dose TA group (300 mg·kg^−1^·d^−1^); Mid, medium-dose TA group (600 mg·kg^−1^·d^−1^); High, high-dose TA group (1200 mg·kg^−1^·d^−1^). For microbiota analyses, *n* = 7 samples per group. Differential taxa were identified using LEfSe with an LDA score threshold > 4.0 and *p* < 0.05.

The LEfSe analysis results indicated that the biomarkers with statistical differences in the Con group included *Ruminococcus_sp_YE281*, Desulfobacterota, Desulfovibrionaceae, Desulfovibrionales, Desulfovibrionia, and *Desulfovibrio*; those in the Low group included Oscillospirales and *unidentified_Ruminococcaceae*; and those in the High group included Lachnospirales, Lachnospiraceae, Clostridia, Firmicutes, *Roseburia*, and *Roseburia_intestinalis,* which were significantly more abundant than those in the other treatment groups. In contrast, the Mid group showed no differentially abundant biomarkers across the four treatment groups. These results were consistent with the findings of the species composition analysis (Figure 7A,B).

#### 3.6.4. KEGG Pathway Analysis

**Figure 8:**
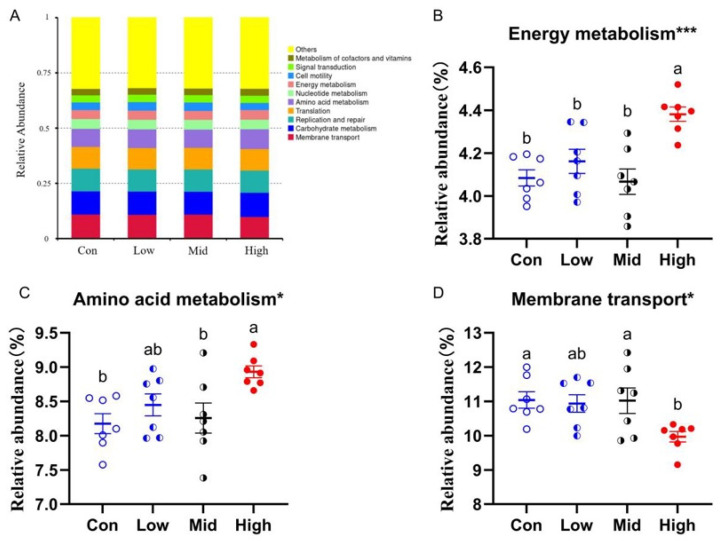
Effects of TA on predicted gut microbiota functional profiles of Brandt’s voles. (**A**) Relative abundance of predicted gut microbiota functional pathways. (**B**) Predicted energy metabolism. (**C**) Predicted amino acid metabolism. (**D**) Predicted membrane transport. Different letters above the columns indicate significant differences. Con, control group; Low, low-dose TA group (300 mg·kg^−1^·d^−1^); Mid, medium-dose TA group (600 mg·kg^−1^·d^−1^); High, high-dose TA group (1200 mg·kg^−1^·d^−1^). For microbiota analyses, *n* = 7 samples per group. Group differences were assessed using one-way ANOVA with Tukey’s post hoc test. One star (*) indicates 0.01 ≤ *p* < 0.05, three stars (***) indicate 0.0001 ≤ *p* < 0.001.

Tax4Fun was used to perform predictive KEGG functional analysis on all samples. The results revealed that the predominant predicted functions in the cecal samples of Brandt’s voles included membrane transport, carbohydrate metabolism, replication and repair, translation, amino acid metabolism, nucleotide metabolism, energy metabolism, cell motility, signal transduction, and cofactor and vitamin metabolism (Figure 8A). The High group exhibited significantly higher predicted amino acid metabolism (F_3,24_ = 4.477, *p* = 0.012) than the Con and Mid groups; significantly elevated predicted energy metabolism (F_3,24_ = 9.090, *p* < 0.001) compared to the Con, Low, and Mid groups; and conversely, significantly lower predicted membrane transport (F_3,24_ = 3.764, *p* = 0.024) than in the Con and Mid groups (Figure 8B–D).

#### 3.6.5. Correlation Between Gut Microbiota and Immunity

**Figure 9:**
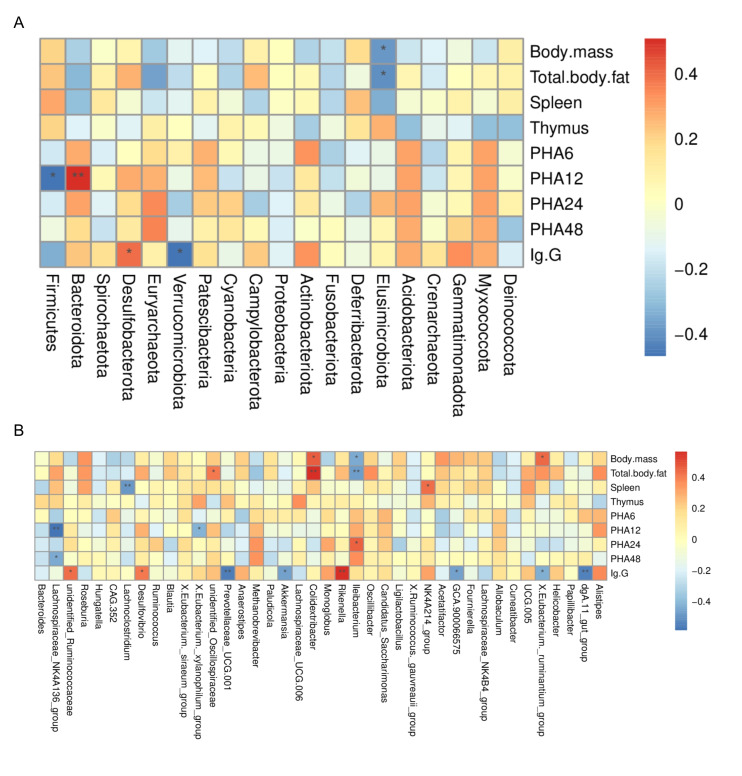
Correlations of gut microbiota with physiological and immune indices in Brandt’s voles at the phylum (**A**) and genus (**B**) levels. * *p* < 0.05, ** *p* < 0.01. Con, control group; Low, low-dose TA group (300 mg·kg^−1^·d^−1^); Mid, medium-dose TA group (600 mg·kg^−1^·d^−1^); High, high-dose TA group (1200 mg·kg^−1^·d^−1^).

At the phylum level, Elusimicrobiota abundance was negatively associated with both body mass and total fat mass. Regarding immune parameters, Bacteroidota abundance was positively associated with PHA12, whereas Firmicutes abundance exhibited a negative correlation. Furthermore, serum IgG titer was positively associated with Desulfobacterota abundance but negatively associated with Verrucomicrobiota abundance (Figure 9A).

The top 40 genera and their correlations with the physiological and immune indices of Brandt’s voles are presented in the figure. Specifically, *Colidextribacter* and *[Eubacterium]_ruminantium_group* were positively associated with body mass, while *Ileibacterium* was negatively associated with this trait. *Unidentified_Oscillospiraceae* and *Colidextribacter* were positively associated with total fat weight, whereas *Ileibacterium* was negatively associated with this index. The *NK4A214_group* was positively associated with spleen weight, while *Lachnoclostridium* was negatively associated with this parameter. *Lachnospiraceae_NK4A136_group* and *[Eubacterium]_xylanophilum_group* were negatively associated with PHA12, *Ileibacterium* was positively associated with PHA24, and *Lachnospiraceae_NK4A136_group* was negatively associated with PHA48. *Unidentified_Ruminococcaceae*, *Desulfovibrio*, and *Rikenella* were positively associated with IgG titer. In contrast, *Prevotellaceae_UCG-001*, *Akkermansia*, *GCA-900066575*, *[Eubacterium]_ruminantium_group*, and *dgA-11_gut_group* were negatively associated with this index (Figure 9B).

## 4. Discussion

### 4.1. Effects of TA on Body Weight and Body Composition

Regarding body mass, previous studies have reported divergent effects of TA on the animal growth performance: the TA treatment reduced body mass in Brandt’s voles [20,27,47] and broiler chickens [48,49], potentially via four mechanisms: (i) forming tannin–salivary protein complexes that elicit astringency and reduce feeding willingness [50,51,52,53]; (ii) modulating satiety hormones (e.g., cholecystokinin) to decrease food intake [54]; (iii) inhibiting fatty acid synthase to suppress adipocyte differentiation [55]; and (iv) suppressing adipogenesis and inducing adipocyte apoptosis [54]. Conversely, TA was shown to increase body mass in Brandt’s voles [26] and hybrid sturgeons [56] while having no effect on adolescent or adult Brandt’s voles [12,23,57] or piglets [16,58]. Consistent with the present study, different concentrations of TA treatment did not significantly affect body mass in Brandt’s voles. Possible reasons include (i) physiological adaptation mechanisms (e.g., increased water intake) [59], modified small intestinal morphology [60], enhanced salivary protein secretion [22,61]) that counteract TA-induced feeding inhibition and re-establish feeding homeostasis; (ii) intestinal lumen expansion to maintain body mass and nutrient absorption efficiency [60]; and (iii) sex-specific tolerance, as female voles exhibit lower sensitivity to TA exposure [62]. In addition, daily food intake and water consumption were not quantitatively monitored in this study. As TA is known to influence palatability, feeding behavior, and digestive efficiency, the absence of these measurements limits our ability to distinguish whether the observed maintenance of body mass reflects compensatory changes in intake and absorption efficiency. Future studies incorporating continuous monitoring of feeding and drinking behavior will be important for clarifying the mechanisms underlying TA-mediated effects on growth and energy balance.

Consistent with the alterations in body mass, TA treatment had no effect on the mass of major adipose tissues (including perigonadal, mesenteric, retroperitoneal, and subcutaneous fat) or total fat mass in Brandt’s voles. Although no statistically significant differences were detected, a consistent trend toward lower fat mass was observed across TA-treated groups, which is directionally consistent with previous reports showing significant reductions in adipose tissue mass following TA treatment in Brandt’s voles and Wistar rats [63,64]. These findings suggest that TA may have modest effects on lipid metabolism, but these effects did not reach statistical significance under the current experimental conditions. Due to inter-individual variability and the limited sample size, fat-related parameters exhibited substantial between-subject variation, and effects of small magnitude may not have been detectable. Future studies should incorporate larger sample sizes to increase statistical power, thereby allowing more robust and reliable findings to be identified.

### 4.2. Effect of TA on Immunity

With respect to cellular immunity, no significant differences in footpad thickness were observed among groups at 6, 12, 24, or 48 h after PHA injection, suggesting that TA may not impair cell-mediated immune function in Brandt’s voles. This result is consistent with findings in sheep [65] but differs from those reported in broiler chickens [66], which may reflect interspecific variation. Notably, the PHA-induced swelling response represents only one proxy of cell-mediated immunity, and other immune pathways may still be affected. Therefore, further studies incorporating additional immune biomarkers and pathways are warranted to comprehensively evaluate the effects of TA on cell-mediated immune function.

With respect to humoral immunity, a dose-dependent decline in serum anti-KLH IgG titers was observed in Brandt’s voles. Relative to the control, IgG titers were reduced by 5.52% in the low-dose group (300 mg·kg^−1^·d^−1^), 18.01% in the medium-dose group (600 mg·kg^−1^·d^−1^), and 55.66% in the high-dose group (1200 mg·kg^−1^·d^−1^). Notably, IgG titers in the high-dose group (1200 mg·kg^−1^·d^−1^) were significantly lower than those in the other three groups, suggesting that a putative threshold may have been exceeded at the high dose [67] and that humoral immune function may have been compromised in Brandt’s voles, which is consistent with previous work [20]. Two potential mechanisms may underlie this phenomenon: (i) TA inhibits IgG production [68]; (ii) TA interacts with IgG to form insoluble precipitated complexes [69].

As important immune organs, the thymus and spleen have weights that serve as key indicators for evaluating changes in animal immune function [70]. The results of the present study revealed that different concentrations of TA did not exert significant effects on the wet masses of the thymus or spleen in Brandt’s voles. This finding is consistent with previous studies demonstrating that different TA doses do not affect immune organ weights in broiler chickens [17,66]. Additionally, adipose tissue has recently been recognized as an immune organ, and reductions in body fat mass can impair immune capacity [71,72]. In the present study, TA treatment did not affect the fat content of Brandt’s voles. The immune functions associated with adipose tissue may be preserved. However, because fat-content-associated immune markers were not directly assessed, future work should further quantify fat-related immune markers, such as cytokine expression and immune cell infiltration, to evaluate potential alterations in immune function within adipose tissue. Collectively, these findings suggest that TA treatment may exert differential effects on specific components of the immune system.

### 4.3. Effects of TA on Gut Microbiota

The analysis of the gut microbiota revealed that the TA treatment significantly altered the composition and structure of the intestinal microbiota in Brandt’s voles at the phylum, family, and genus levels. TA treatment at 1200 mg·kg^−1^·d^−1^ markedly reduced the relative abundance of Desulfobacterota and its associated taxa, including Desulfovibrionia, Desulfovibrionales, Desulfovibrionaceae, and *Desulfovibrio*. Desulfobacterota represents a group of potentially harmful intestinal bacteria, whose primary metabolic product, hydrogen sulfide (H_2_S), exerts toxic effects on intestinal epithelial cells. Furthermore, previous studies have demonstrated that increased Desulfobacterota relative abundance is closely associated with metabolic diseases, such as obesity [73,74]. *Desulfovibrio* can disrupt the intestinal epithelial barrier by producing hydrogen sulfide and activating the TLR4 signaling pathway, thereby exacerbating inflammatory responses [75,76]. Meanwhile, TA at 1200 mg·kg^−1^·d^−1^ significantly increased the relative abundance of Firmicutes and its affiliated taxa at lower taxonomic levels, including Clostridia, Lachnospirales, Lachnospiraceae, *Roseburia*, and *Roseburia intestinalis*. Firmicutes is functionally heterogeneous, with some genera being potentially beneficial, such as Lachnospirales, which are capable of producing short-chain fatty acids (SCFAs), such as acetic acid and butyric acid, which provide energy to the host and help maintain intestinal barrier integrity [47,77]. *Roseburia*, a genus of beneficial gut bacteria, enhances intestinal barrier function and alleviates inflammation, and its relative abundance is negatively correlated with body weight [73,78]. However, not all Firmicutes members are beneficial, and some can be associated with negative effects on health depending on the specific genera present. As one of the dominant gut bacterial phyla, Firmicutes plays a crucial role in promoting fiber degradation and improving energy utilization efficiency; increases in its relative abundance are generally considered to enhance the host’s capacity to acquire energy from the diet [79,80,81]. Notably, the increased relative abundance of Firmicutes observed in the present study is inconsistent with the findings of Gu [47], who reported that TA-induced weight loss in Brandt’s voles was accompanied by a decrease in Firmicutes richness and suggested that this weight loss was associated with a TA-induced reduction in Firmicutes. However, in the current study, despite a significant increase in the relative abundance of Firmicutes in the high-dose TA treatment group (1200 mg·kg^−1^·d^−1^), no significant change in body weight was observed.

With respect to the overall gut microbiota structure, TA significantly altered the intestinal microbial structure of Brandt’s voles, suggesting a potential dual regulatory pattern characterized by an increase in taxa commonly associated with beneficial functions and a decrease in taxa often linked to adverse effects. Such alterations in microbiota composition may influence host physiological status through multiple pathways: the increased abundance of *Roseburia* may enhance intestinal barrier function and alleviate inflammatory responses, whereas the reduced relative abundance of Desulfobacterota may lower the risk of intestinal mucosal damage induced by hydrogen sulfide. These results are consistent with the findings of Shi [82]. However, they are inconsistent with previous studies reporting that high-dose TA (1200 mg·kg^−1^·d^−1^) reduced the relative abundance of beneficial bacteria and increased the relative abundance of pathogenic bacteria in Brandt’s voles [27,47]. This discrepancy may be related to the sex of the experimental animals and the duration of treatment in the present study.

Furthermore, high-dose TA (1200 mg·kg^−1^·d^−1^) significantly altered the species richness and diversity of the intestinal microbiota in Brandt’s voles. The Observed features, Chao1, and Shannon indices of the gut microbiota in the High group were significantly lower than those in the Con and Low groups. These findings demonstrate that intragastric administration of 1200 mg·kg^−1^·d^−1^ of TA significantly altered the alpha diversity of the cecal microbiota and reduced the diversity and richness of the intestinal microbiota in Brandt’s voles. While a reduction in diversity is often considered indicative of microbial community imbalance, it is important to recognize that lower diversity is not always detrimental. Reduced diversity can sometimes reflect a more stable or resilient microbial community, depending on the context. These findings indicate that TA can modulate the enrichment level of the cecal microbiota in Brandt’s voles, thereby reshaping the structure of the cecal microbiota. Compared with the control group, the High group exhibited significant changes in the beta diversity of the cecal microbial community, indicating that intragastric administration of high-concentration TA altered the intestinal microbial community structure of Brandt’s voles. No significant differences were observed between the control group and the low (300 mg·kg^−1^·d^−1^)- or medium-dose (600 mg·kg^−1^·d^−1^) TA treatment groups, suggesting that the effects of TA on the cecal microbiota of Brandt’s voles are dose-dependent. Predictive KEGG functional analysis revealed that different concentrations of TA treatment significantly influenced the predictive functional pathways of the cecal microbiota in Brandt’s voles, exhibiting a clear dose-dependent effect. Intragastric administration of TA at 1200 mg·kg^−1^·d^−1^ significantly increased the overall relative abundance of predictive pathways related to amino acid metabolism and energy metabolism while decreasing the predictive functions associated with membrane transport. These results indicate a potential shift in the functional profile of the cecal microbiota, from a pattern dominated by substrate uptake-related pathways to one focused on internal metabolic processes [83]. These predictive results suggest a possible association between TA-induced alterations in microbial functional profiles and host physiological processes; however, this relationship remains exploratory and requires validation through direct functional and metabolic measurements. Future studies using targeted metabolite profiling and multi-omics approaches will be necessary to validate these predicted functional patterns and clarify their links to host physiology [84]. These findings provide a basis for further research on the role of intestinal microbiota in the health, environmental adaptation, and metabolism of Brandt’s voles.

### 4.4. Relationship Between Immunity and Gut Microbiota

In the gut microbiota, microbial community members play a crucial role in regulating host inflammation and immune homeostasis. Correlation analysis in the present study revealed that Bacteroidota abundance was positively correlated with footpad thickness in voles 12 h after PHA injection. This finding suggests a possible link between Bacteroidota and immune activation in Brandt’s voles, consistent with previous studies demonstrating the pro-inflammatory effects of Bacteroides [85]. In contrast, the relative abundance of Firmicutes was negatively correlated with footpad thickness at 12 h after PHA injection, suggesting a potential association with an anti-inflammatory role. This is consistent with the known ability of Firmicutes to produce anti-inflammatory metabolites, such as short-chain fatty acids (SCFAs). Additionally, Desulfobacterota abundance was positively correlated with IgG titers, and its relative abundance was reduced in the High group. These results suggest that high-dose TA (1200 mg·kg^−1^·d^−1^) may influence immune regulation, possibly through the suppression of Desulfobacterota.

At the genus level, *Desulfovibrio* exhibited a positive correlation with IgG titers, a finding that suggests this genus may promote inflammatory responses, which is consistent with the results of previous research [75]. Members of *Lachnospiraceae* and *Rikenella* have been demonstrated to ferment dietary fibers and produce butyric acid [86]. *Rikenella* was positively correlated with IgG titers, whereas *Lachnospiraceae_NK4A136_group* was negatively correlated with PHA48. These results indicate that the gut microbiota may collectively regulate immune homeostasis through functional specialization. Additionally, *Ileibacterium* was positively correlated with PHA24, suggesting that it may be involved in cellular immunity activation. The *NK4A214_group* was positively correlated with spleen mass, whereas *Lachnoclostridium* was negatively correlated with it. Together, these findings further highlight potential associations among different microbial taxa and host immune regulation, indicating that the intestinal microbial community may contribute to maintaining host immune homeostasis through complex network interactions.

Several limitations of the present study warrant consideration. First, the investigation focused exclusively on adult females. Given that foraging behavior [87], endocrine profiles [88], and immune resource allocation [89] may differ between sexes, our findings are primarily representative of adult female Brandt’s voles under chronic, controlled TA exposure; therefore, extrapolating these results to males or heterogeneous wild populations should be approached with caution. Second, while standardized gavage protocols were strictly followed across all groups, there was a lack of a sham-gavaged control group to distinguish TA effects from handling/gavage stress. Future work should examine sex × dose interactions using matched male and mixed-sex designs, and incorporate sham or no-gavage controls and, where feasible, objective stress markers to better quantify procedural contributions.

## 5. Conclusions

In summary, this study shows that tannic acid at 1200 mg·kg^−1^·d^−1^ is associated with distinct shifts in the gut microbiota structure of Brandt’s voles, characterized by reduced microbial diversity and changes in the relative abundance of specific taxa. These microbiota alterations co-occur with a dose-dependent suppression of a specific humoral immune index (anti-KLH IgG), while other growth-related and immune parameters remain largely unchanged. The observed patterns suggest a potential role of the microbiota–immune axis in host responses to dietary TA; however, the underlying mechanisms require further investigation. Future studies, including targeted microbial isolation, functional validation, and fecal microbiota transplantation (FMT), are necessary to test these hypotheses and to establish causal relationships between microbiota alterations and host immune and metabolic outcomes.

## Figures and Tables

**Table 1:** Effects of tannic acid on body composition in Brandt’s voles.

Parameters	Con Group	Low Group	Mid Group	High Group	Statistical Summary		
					F_3,29_	*p*	η^2^
Initial body mass (g)	43.4 ± 1.8	44.0 ± 2.3	43.4 ± 1.2	45.0 ± 1.3	0.448	0.720	0.044
Final body mass (g)	46.4 ± 3.9	48.2 ± 4.1	44.7 ± 2.5	48.2 ± 2.1	0.311	0.817	0.031
Wet carcass mass (g)	34.5 ± 3.5	35.8 ± 3.3	31.9 ± 2.2	35.8 ± 2.2	0.454	0.716	0.045
Perigonadal fat (g)	0.649 ± 0.285	0.559 ± 0.288	0.292 ± 0.103	0.460 ± 0.121	0.582	0.631	0.057
Perigonadal fat content (%)	1.566 ± 0.469	1.341 ± 0.545	0.800 ± 0.247	1.169 ± 0.286	0.733	0.541	0.070
Mesenteric fat (g)	0.317 ± 0.060	0.253 ± 0.042	0.245 ± 0.031	0.243 ± 0.031	0.701	0.559	0.068
Mesenteric fat content (%)	0.902 ± 0.112	0.699 ± 0.065	0.759 ± 0.065	0.671 ± 0.059	1.738	0.181	0.152
Retroperitoneal fat (g)	0.877 ± 0.376	0.540 ± 0.128	0.434 ± 0.177	0.616 ± 0.163	0.670	0.578	0.065
Retroperitoneal fat content (%)	2.129 ± 0.643	1.427 ± 0.216	1.181 ± 0.426	1.571 ± 0.396	0.782	0.514	0.075
Subcutaneous fat (g)	1.444 ± 0.560	1.165 ± 0.834	0.726 ± 0.141	0.783 ± 0.109	1.155	0.344	0.107
Subcutaneous fat content (%)	3.583 ± 0.922	3.057 ± 0.532	2.131 ± 0.301	2.107 ± 0.206	1.848	0.161	0.160
Total body fat (g)	3.729 ± 1.360	2.897 ± 0.837	2.019 ± 0.471	2.101 ± 0.403	0.965	0.423	0.091
Total body fat content (%)	9.379 ± 2.116	7.548 ± 1.380	5.856 ± 1.021	5.518 ± 0.867	1.659	0.198	0.146

Values are presented as the mean ± SE. Body composition parameters were analyzed by one-way ANOVA followed by Tukey’s post hoc test. Con, control group; Low, low-dose TA group (300 mg·kg^−1^·d^−1^); Mid, medium-dose TA group (600 mg·kg^−1^·d^−1^); High, high-dose TA group (1200 mg·kg^−1^·d^−1^).

## Data Availability

All data analyzed during this study are included in the published article.

## Supplementary Materials

The following supporting information can be downloaded at: https://www.mdpi.com/article/10.3390/microorganisms14030577/s1, Figure S1: Relative abundance of gut microbiota composition in Brandt’s voles across treatment groups at the family (A) and genus (B) levels. Figure S2: Effects of TA on gut microbiota composition in Brandt’s voles. Differences in relative abundance of gut microbiota at the family (A–E) and genus (F–M) levels among different TA treatment groups. Table S1: Pairwise beta diversity statistics based on Bray–Curtis distances among treatment groups.
