# Supplementary material for: Effects of Tannic Acid on Immune Function and Gut Microbiota in Brandt’s Voles (Lasiopodomys brandtii)
Source: Microorganisms. 2026 Mar 3;14(3):577. doi: 10.3390/microorganisms14030577 (PMC13028715; doi:10.3390/microorganisms14030577)
Supplement: Supplementary file 1 [file microorganisms-14-00577-s001.zip › microorganisms-4087312-supplementary.pdf]

Supplementary

Effects of Tannic Acid on Immune Function and Gut Microbiota in Brandt's Voles (*Lasiopodomys brandtii*)

Jin Li, Kunying Zhou, Di Xu, Yunqi Liu, Yu Sun and Deli Xu *

School of Life Sciences, Qufu Normal University, Qufu 273165, China; 17861824275@163.com (J.L.); zky7665@163.com (K.Z.); xdvin458@163.com (D.X.); 17562002639@163.com (Y.L.); 13555316428@163.com (Y.S.)

* Correspondence: xudl1975@163.com. ORCID: 0000-0002-1515-2985.

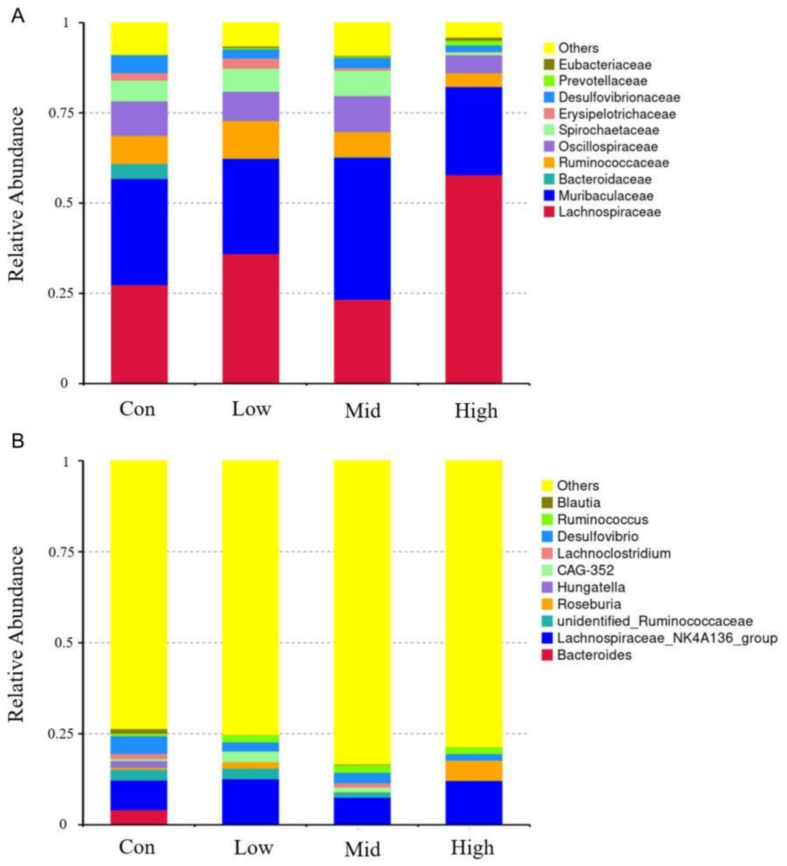

Figure S1. Relative abundance of gut microbiota composition in Brandt's voles across treatment groups at the family (A), and genus (B) levels. Con, control group; Low, low-dose TA group; Mid, medium-dose TA group; High, high-dose TA group. For microbiota analyses, n = 7 samples per group.

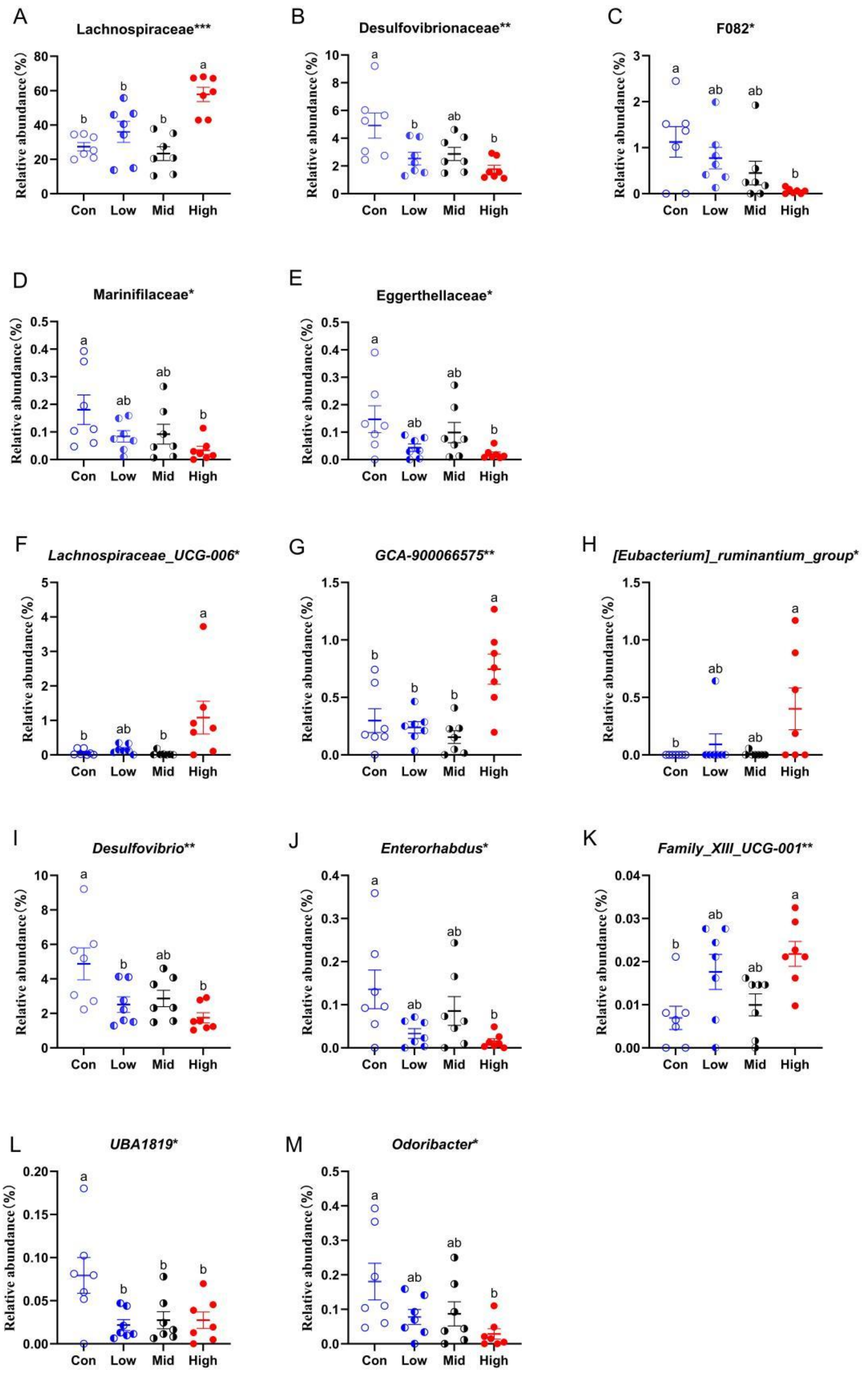

Figure S2. Effects of TA on gut microbiota composition in Brandt's voles. Differences in gut microbiota relative abundance at the family (A–E), and genus (F–M) levels among different TA treatment groups. Different letters above the columns indicate statistical significance among the groups(* $p < 0.05$, ** $p < 0.01$). Con, control group; Low, low-dose TA group; Mid, medium-dose TA group; High, high-dose TA group. For microbiota analyses, n = 7 samples per group.

Table S 1. Pairwise beta diversity statistics based on Bray–Curtis distances among treatment groups.

Comparison	PERMANOVA F	PERMANOVA R^2	PERMANOVA P	ANOSIM R	ANOSIM P
Con vs Low	1.018	0.078	0.418	−0.035	0.650
Con vs Mid	0.957	0.074	0.541	−0.035	0.706
Con vs High	2.433	0.169	<0.001	0.538	0.002
Low vs Mid	0.918	0.071	0.701	−0.027	0.546
Low vs High	2.294	0.160	<0.001	0.658	0.001
Mid vs High	2.074	0.147	<0.001	0.492	0.001

Beta diversity was assessed using pairwise PERMANOVA (Adonis) and ANOSIM. PERMANOVA R^2 indicates the proportion of variance explained by treatment, and ANOSIM R reflects the degree of group separation. P values were obtained by permutation testing. Con, control group; Low, low-dose TA group; Mid, medium-dose TA group; High, high-dose TA group. For microbiota analyses, n = 7 samples per group.
